# Clinical utility of repeated rebiopsy for EGFR T790M mutation detection in non-small cell lung cancer

**DOI:** 10.3389/fonc.2024.1452947

**Published:** 2024-08-26

**Authors:** Eun Hye Lee, Se Hyun Kwak, Kyeong Yeon Kim, Chi Young Kim, Sang Hoon Lee, Seok-Jae Heo, Yoon Soo Chang, Eun Young Kim

**Affiliations:** ^1^ Division of Pulmonology, Allergy and Critical Care Medicine, Department of Internal Medicine, Yongin Severance Hospital, Yonsei University College of Medicine, Gyeonggi-do, Republic of Korea; ^2^ Department of Internal Medicine, Gangnam Severance Hospital, Yonsei University College of Medicine, Seoul, Republic of Korea; ^3^ Division of Pulmonary and Critical Care Medicine, Department of Internal Medicine, Severance Hospital, Yonsei University College of Medicine, Seoul, Republic of Korea; ^4^ Division of Biostatistics, Department of Biomedical Systems Informatics, Yonsei University College of Medicine, Seoul, Republic of Korea

**Keywords:** lung cancer, EGFR, T790M, rebiopsy, repeated biopsy

## Abstract

**Purpose:**

In cases where rebiopsy fails to find the epidermal growth factor receptor (EGFR) T790M mutation, the criteria for selecting patients for repeated rebiopsy remains unclear. This study aimed to assess the impact of repeated rebiopsy on T790M mutation detection in non-small cell lung cancer (NSCLC) patients.

**Methods:**

Patients with advanced EGFR-mutated NSCLC between January 2018 and December 2021 at three-referral hospitals in South Korea underwent retrospective review. Of 682 patients who had rebiopsy after disease progression, T790M mutation status was assessed in plasma circulating tumor DNA (ctDNA) and/or tumor tissues.

**Results:**

The overall T790M positivity rate increased from 40.8% after the first rebiopsy to 52.9% following multiple rebiopsies in the entire study population. Longer duration of initial EGFR TKI use (OR 1.792, ≥8 months vs. <8 months, *p=*0.004), better EGFR TKI responses (OR 1.611, complete or partial response vs. stable disease, *p*=0.006), presence of bone metastasis (OR 2.286, *p*<0.001) were correlated with higher T790M positivity. Longer EGFR TKI use and better responses increased T790M positivity in repeated tissue rebiopsy, while bone metastasis favored liquid rebiopsy. Additionally, T790M status has been shown to be positive over time through repeated rebiopsies ranging from several months to years, suggesting its dynamic nature.

**Conclusion:**

In this study, among patients who initially tested negative for T790M in rebiopsy, repeated rebiopsies uncovered an additional 23.5% T790M positivity. Particularly, it is suggested that repeated rebiopsies may be valuable for patients with prolonged EGFR TKI usage, better responses to treatment, and bone metastasis.

## 1 Introduction

Lung cancer is the second most frequently diagnosed cancer, with approximately 2.2 million new cases and 1.8 million deaths worldwide in 2020 (1). It is also the leading cause of cancer-related morbidity and mortality in both men and women in South Korea (2). The development of epidermal growth factor receptor (EGFR)-targeted tyrosine kinase inhibitors (TKIs) has resulted in significant improvements in the treatment outcomes of patients with non-small cell lung cancer (NSCLC) harboring EGFR mutations. Despite remarkable initial responses, most patients experience disease progression after approximately 9–12 months, which is attributed to the development of acquired resistance to EGFR-TKIs (3–5). Several mechanisms for EGFR-TKI resistance have been proposed, including the amplification of MET and ERBB2, activation of downstream MAPK or PI3K pathways, and small cell lung cancer transformation. Among these, the most prevalent acquired resistance mechanism is the Thr790Met (T790M) point mutation in exon 20, which accounts for approximately 50–60% of all resistance cases (6, 7). Most recently, third-generation EGFR-TKIs, such as osimertinib and lazertinib, have been used to overcome this resistance mechanism. Therefore, accurate detection of the acquired EGFR T790M mutation is crucial as it can guide the use of third-generation EGFR-TKIs in patients with this mutation, leading to potential benefits.

Previous studies had suggested that the EGFR T790M mutation, although not detected in initial rebiopsies, can be found through repeated rebiopsies (8, 9). In a recent meta-analysis by Kim et al. (10) reported that approximately 10.3% of patients were found to have additional T790M mutation through repeated rebiopsies. However, repeated rebiopsies entail increased risks and costs, making it a significant challenge to determine which patients could benefit from additional rebiopsies in clinical practice. In this study, we aimed to examine the clinical features affecting the T790M positivity and to identify the characteristics of patient groups most likely to benefit from subsequent rebiopsies.

## 2 Materials and methods

### 2.1 Participants

**Figure 1:**
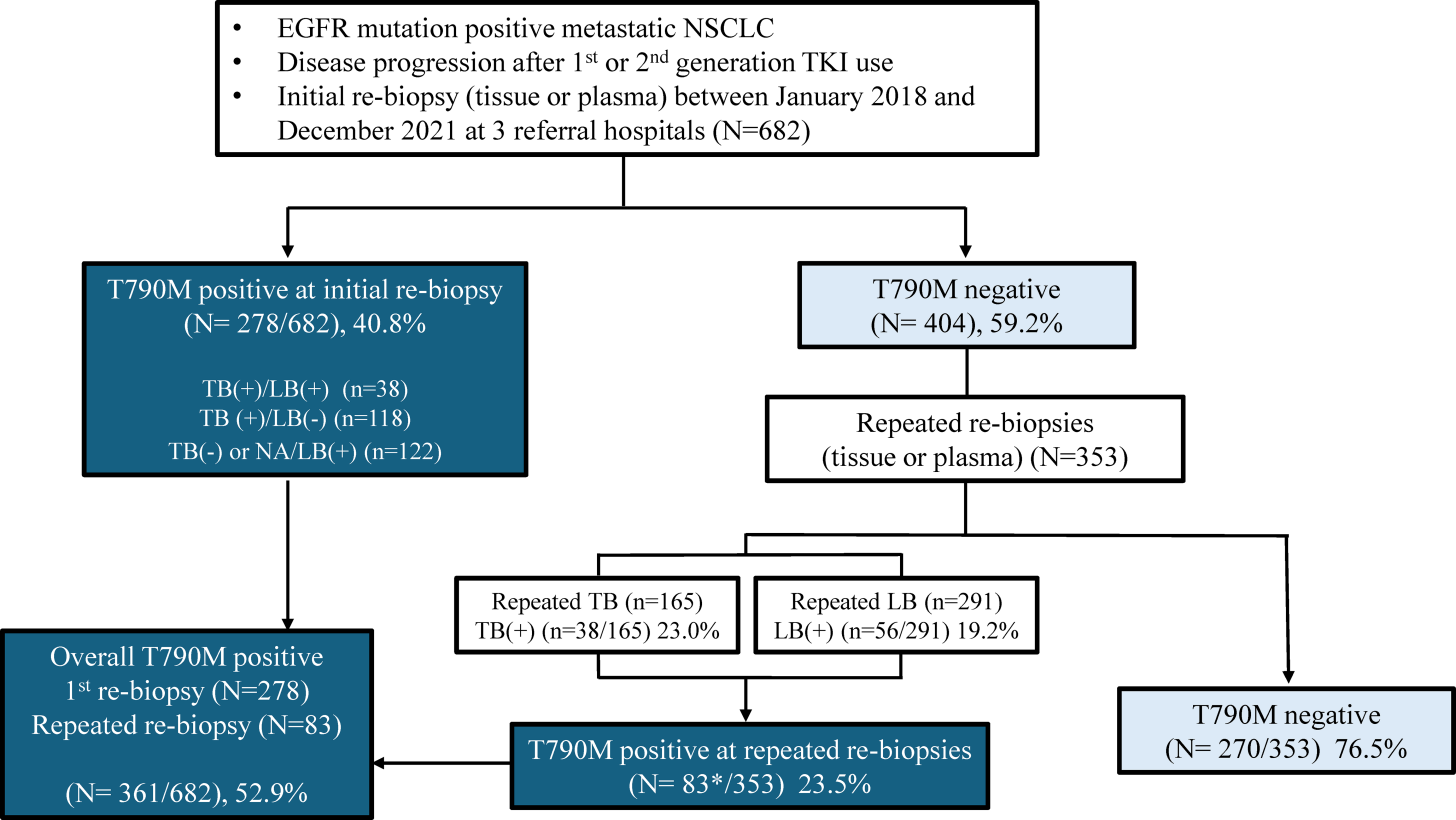
Schematic diagram of study patients. *11 patients showed positive T790M results in both tissue and liquid samples from repeated rebiopsies. NA, not available; TB, tissue biopsy; LB, liquid biopsy.

Patients with advanced EGFR mutated NSCLC between January 2018 and December 2021 at three-referral hospitals (Severance Hospital, Gangnam Severance Hospital, and Yongin Severance Hospital) were retrospectively reviewed. Final analysis was conducted on 682 patients who had disease progression with prior first- or second-generation EGFR inhibitors (**Figure 1**). This retrospective study was approved by the Institutional Review Board of our hospital, and the requirement for informed consent was waived (IRB No. 9-2021-0188).

### 2.2 Measurements

We retrospectively reviewed the medical records for patient demographics, types of EGFR-TKIs used, EGFR mutation types, number and timing of rebiopsies, and the presence of the T790M mutation. The evaluation of the response to TKI treatment was assessed using the RECIST criteria version 1.1. We evaluated progression-free survival (PFS) from the first day of TKI treatment to disease progression or death. We defined the objective response rate (ORR) as the percentage of patients who achieved at least one complete response (CR) or partial response (PR) prior to any evidence of progression. The disease control rate (DCR) was defined as the percentage of patients who achieved stable disease (SD) or CR, or PR.

### 2.3 Detection of T790M mutation

All included patients underwent EGFR mutation testing after cancer progression through tissue or plasma (liquid) biopsy. The T790M mutation status was assessed in the plasma ctDNA and tissues. For tissue biopsy, EGFR PNA Mutyper™ R Detection Kit (Panagene, Daejeon, South Korea) or GenesWell™ ddEGFR Mutation Test v1 (Gencurix, Seoul, South Korea) was performed according to the manufacturer’s instructions. For plasma biopsy, the Cobas**^®^** EGFR Mutation Test v2 (Roche Molecular Systems, CA, USA) was used in all three hospitals.

### 2.4 Statistical analysis

Continuous variables are presented as the mean ± standard deviation (SD) or median and interquartile range (IQR) and were analyzed using Student’s *t*-test or Mann–Whitney test. Categorical variables are reported as numbers and percentages and were analyzed using the chi-square test or Fisher’s exact test. Clinical factors associated with T790M positivity were investigated using logistic regression models, and the results are reported as odds ratios (ORs) and 95% confidence intervals (CIs). The T790M results from repeated tissue and liquid rebiopsies were illustrated using a swimmer plot. We used Kaplan Meier methods with log-rank test for survival analysis and to estimate the median time to event, including 95% CI. All tests of significance were two-tailed, and statistical significance was set at p value < 0.05. Statistical analysis was performed using the R software (version 4.1.1; R Foundation for Statistical Computing, Vienna, Austria).

## 3 Results

### 3.1 Baseline characteristics of study patients

The baseline characteristics of the patients in this study are listed in **Table 1**. The median age was 62 years, and 62.5% of the patients were women. Of these, 74.6% had never smoked. CNS metastases were observed in 34.2% of the patients, whereas bone metastases were present in 34.9% of the patients. The median duration of the first or second generation TKI use was 14 months. After disease progression, the overall rate of positive T790M mutations either tissue or liquid rebiopsies was 52.9% (361/682). Among a total of 531 patients with tissue rebiopsies, 165 patients (accounting for 31.1%) underwent repeated rebiopsies more than two times. For liquid rebiopsies, repeated rebiopsies were performed two or more times in 291 patients, corresponding to 42.7% (**Table 1**).

**Table 1:** Baseline and clinical characteristics of study patients.

Characteristic	Total (N=682)
Age, median (IQR)	62 (54-69)
≥65 years, n (%)	273 (40.0)
<65 years, n (%)	409 (60.0)
Sex, n (%)	
Male	225 (37.8)
Female	424 (62.2)
BMI, mean ± SD	23.1 ± 2.95
Smoking history, n (%)	
Never smoked	509 (74.6)
Former smoker	158 (23.2)
Current smoker	15 (2.2)
ECOG PS, n (%)	
0	303 (44.4)
1	365 (53.5)
≥2	14 (2.1)
Histologic type, n (%)	
Adenocarcinoma	593 (94.4)
Squamous carcinoma and others	35 (5.6)
Clinical stage at diagnosis, n (%)	
III	39 (6.2)
IV	589 (93.8)
CNS metastasis at diagnosis, n (%)	233 (34.2)
Liver metastasis at diagnosis, n (%)	55 (8.1)
Bone metastasis at diagnosis, n (%)	238 (34.9)
Prior use of EGFR-TKI, n (%)	
Gefinitib	386 (56.6)
Afatinib	191 (28.0)
Erlotinib	105 (15.4)
Type of EGFR mutation at diagnosis, n (%)	
E19del	374 (54.8)
L858R	254 (37.2)
Others^a^	52 (7.6)
Unknown	2 (0.3)
Median duration of 1st or 2nd TKI use, month (IQR)	14.0 (8.0-25.13)
T790M positive after disease progression, n (%)	
Tissue	207/531 (38.9)
Plasma	218/682 (31.9)
Tissue or plasma	361/682 (52.9)
Patients with Tissue rebiopsy, n (%)	531 (100)
Number of tissue biopsy, once	366 (68.9)
Number of tissue biopsy, twice	110 (20.7)
Number of tissue biopsy ≥3	55 (10.4)
Patients with liquid rebiopsy, n (%)	682 (100)
Number of liquid biopsy test, once	391 (57.3)
Number of liquid biopsy test, twice	186 (27.3)
Number of liquid biopsy test ≥3	105 (15.4)
Survival status at medical record abstraction, n (%)	
Deceased	223 (32.6)
Alive	446 (65.4)
Lost to follow-up	13 (2.1)

a L861Q, S768I, G719X, Ex20Ins.

### 3.2 T790M mutation positivity after 1^st^ rebiopsy and repeated rebiopsies

The presence of T790M mutation in 682 patients who underwent EGFR mutation testing after disease progression with first- or second-generation TKI use are shown in **Figure 1**. All 682 patients had liquid EGFR results whereas 531 patients underwent tissue rebiopsy. In the first rebiopsy tested from either tissue or liquid, the T790M positivity rate was 40.8%, with 278 individuals. Through repeated rebiopsies, an additional 83 positive patients were identified, finally showing a T790M positivity rate of 52.9% among the patients (**Figure 1**). Among the 404 patients who initially tested negative for T790M in the first rebiopsy, 353 underwent repeated rebiopsy. Out of these 353 patients, 83 (23.5%) were subsequently found to be positive for T790M upon repeated rebiopsy. Specifically, the positive rate for the second rebiopsy is 65 out of 353 (18.4%), the third rebiopsy is 10 out of 110 (9.1%), and the fourth rebiopsy is 5 out of 53 (9.4%), the fifth rebiopsy is 2 out of 25 (8%) (data now shown). Ultimately, the T790M positivity rate increased by approximately 12.1% through repeated rebiopsies. Rebiopsies were performed on patients’ primary lung lesions as well as various metastatic sites. While the T790M positive rates are not consistent between primary and metastatic sites, the rate is higher in pleural fluid compared to other sites, and lower in liver, bone, and brain compared to the primary lung site (**Supplementary Table S1**).

### 3.3 Comparison of patients’ characteristics according to T790M mutation

**Figure 2:**
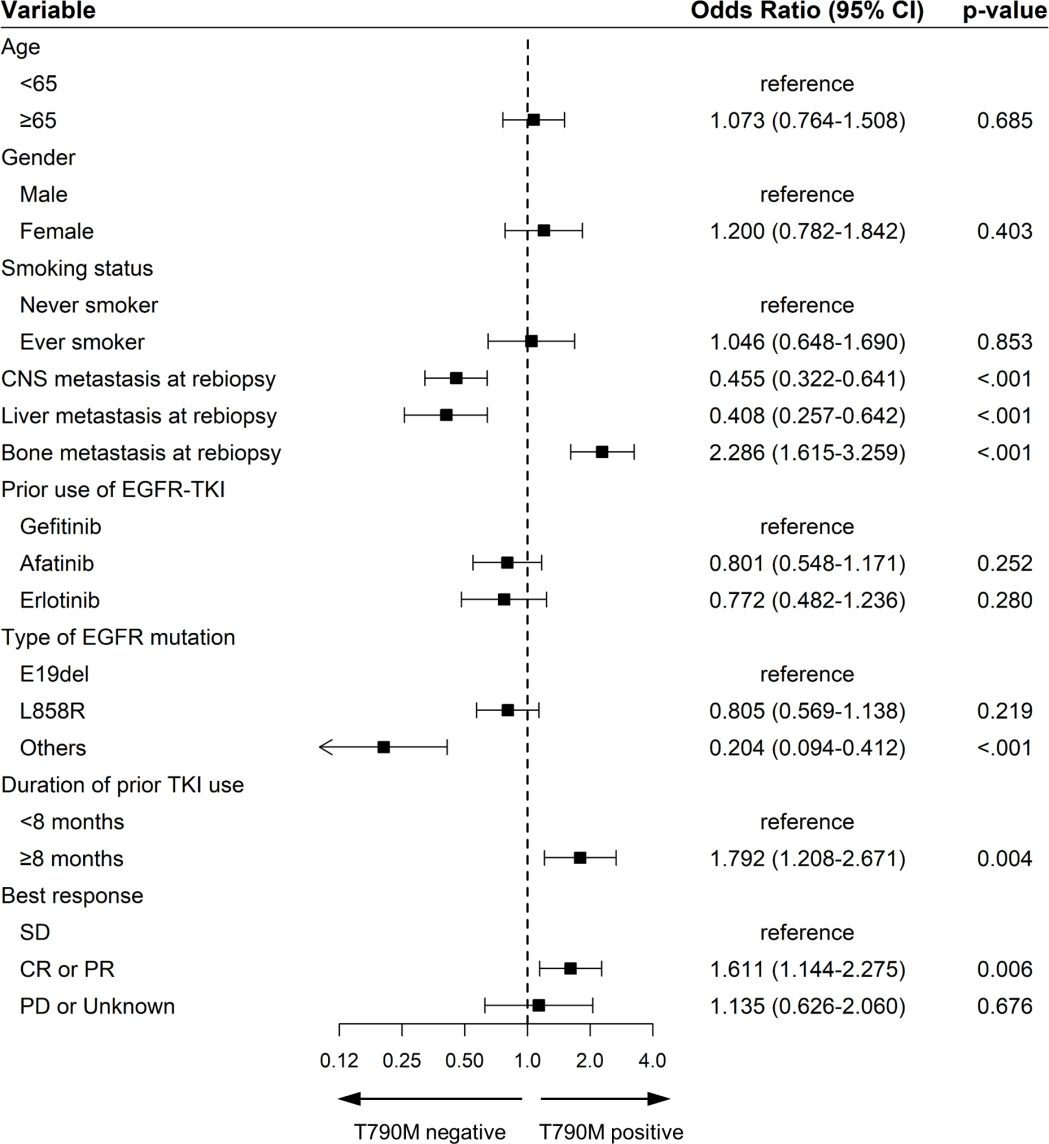
Forest plot for the multivariate analysis of factors for acquired EGFR T790M in patients treated with EGFR-TKIs. Squares represent odds ratios and bars 95% confidence intervals for the odds ratios. OR, odds ratio; 95% CI, 95% confidence interval; EGFR, epidermal growth factor receptor; SD, stable disease; CR, complete response; PR, partial response; PD, progressive disease.

Patient characteristics were compared based on the presence or absence of the T790M mutation on rebiopsy after disease progression (**Table 2**). No significant differences in age, sex, or smoking status were observed between the two groups. However, a significant difference was observed in EGFR TKI treatment response and duration. The T790M-positive group had a longer median duration of previous EGFR inhibitor use (15.0 months vs 12.9 months, *p*=0.011) and better treatment response (CR or PR: 50.9% vs. 39.2%, *p*=0.036) compared to the T790M-negative group. Although there was no significant difference in the rate of extra-thoracic metastasis between the two groups, bone metastasis was significantly higher in the T790M-positive group (54.6% vs. 43.3%, *p=*0.004). Multivariate analysis revealed that the longer duration of initial EGFR TKI use (OR 1.792, with ≥8months compared to <8months, *p*=0.004), better EGFR TKI responses (OR 1.611, with CR or PR compared to SD, *p*=0.006), presence of bone metastasis (OR 2.286, *p*<0.001) were correlates with a higher rate of T790M positivity (**Figure 2**).

**Table 2:** Baseline and clinical characteristics of patients according to T790M mutation.

Characteristic	Total (N=682)	T790M-negative (N=321)	T790M-positive (N=361)	*P* value
Age, median (IQR)	62 (54-69)	61 (55-69)	62 (54-70)	0.884
≥65 years, n (%)	273 (40.0)	127 (39.6)	146 (40.4)	0.815
<65 years, n (%)	409 (60.0)	194 (60.4)	215 (59.6)	
Sex, n (%)				
Male	258 (37.8)	124 (38.6)	134 (37.1)	0.685
Female	424 (62.2)	197 (61.4)	227 (62.9)	
Smoking history, n (%)				
Never smoked	509 (74.6)	239 (74.5)	270 (74.8)	0.084
Ever smoker	173 (25.4)	82 (25.5)	91 (25.2)	
Extra-thoracic metastasis at rebiopsy^a^, n (%)	492 (72.1)	241 (75.1)	251 (69.5)	0.127
CNS metastasis at rebiopsy^a^, n (%)	354 (51.9)	191 (59.5)	163 (45.2)	<0.001
Liver metastasis at rebiopsy^a^, n (%)	89 (13.0)	48 (15.0)	41 (11.4)	0.201
Bone metastasis at rebiopsy^a^, n (%)	336 (49.3)	139 (43.3)	197 (54.6)	0.004
Prior use of EGFR-TKI, n (%)				
Gefitinib	386 (56.6)	166 (51.7)	220 (60.9)	0.053
Afatinib	191 (28.0)	100 (31.2)	91 (25.2)	
Erlotinib	105 (15.4)	55 (17.1)	50 (13.9)	
Type of EGFR mutation at diagnosis, n (%)				
E19del	374 (54.8)	156 (48.9)	218 (60.4)	<0.001
L858R	254 (37.2)	122 (38.2)	132 (36.6)	
Others^b^	52 (7.6)	41 (12.9)	11 (3.0)	
Unknown	2 (0.3)	2 (0.6)	0 (0)	
Median duration of 1st or 2nd TKI use, month (IQR)	14.0 (8.0-25.1)	12.9 (6.6-25.5)	15.0 (9.0-24.2)	0.011
≥8 months, n (%)	527 (77.3)	227 (70.7)	300 (83.1)	0.001
Best Response				
CR	6 (0.9)	3 (0.9)	3 (0.8)	0.036
PR	304 (44.6)	123 (38.3)	181 (50.1)	
SD	311 (45.6)	163 (50.8)	148 (41.1)	
PD or Unknown	61 (8.9)	32 (10.0)	29 (8.0)	

^a^For patients who underwent repeated rebiopsy more than twice, the metastasis site immediately before the final biopsy was reflected. ^b^L861Q, S768I, G719X, Ex20Ins.

### 3.4 Factors affecting T790M positivity in repeated rebiopsy from tissue and plasma

**Supplementary Tables S2**, **S3** showed the characteristics of patients who underwent additional repeated rebiopsies. In **Supplementary Table S2**, the characteristics of patients who underwent more than two repeated tissue rebiopsies were analyzed among 165 individuals, highlighting that a longer median duration of prior TKI use significantly correlated with T790M mutation positivity. In **Supplementary Table S3**, which analyzed 291 patients with repeated liquid rebiopsies, revealing a significant association between T790M positivity and the prevalence of bone metastasis. The multivariate analysis showed that prolonged duration of prior TKI use before rebiopsy (OR 3.203, with ≥8months compared to <8months, *p=*0.049), along with a favorable response to prior TKI treatment (OR 2.805, with CR or PR compared to SD, *p*=0.015), served as predictive indicators for T790M mutation positivity in subsequent tissue rebiopsies (**Supplementary Figure S1**). For individuals undergoing repeated liquid rebiopsies, the occurrence of bone metastasis (OR 2.083, *p*=0.030) was strongly correlated with an increased likelihood of detecting T790M positivity (**Supplementary Figure S2**).

### 3.5 Transition of T790M Status in repeated tissue and liquid biopsies in different time point

**Figure 3:**
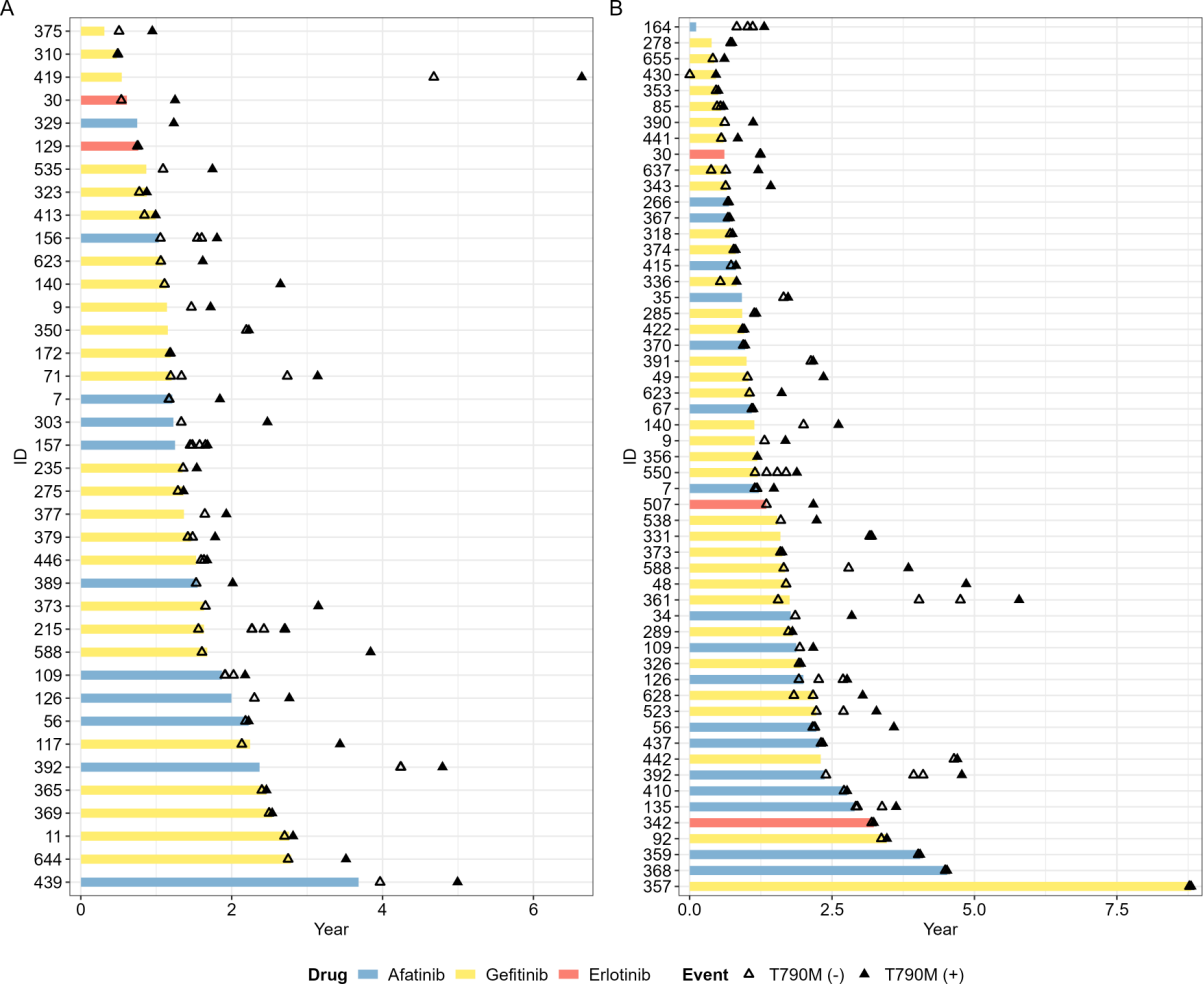
Swimmer plot of EGFR T790M mutation status in repeated tissue and liquid rebiopsies in different time point. **(A)** 38 patients who showed positive EGFR T790M results from repeated tissue rebiopsies after disease progression, and **(B)** 56 patients who showed EGFR T790M positivity from repeated liquid rebiopsies. Empty triangles represent T790M negativity, while filled black triangles indicate T790M mutation positivity. The timing of discontinuation of 1st line TKI treatment coincided with the point of disease progression in most patients.

**Figure 3** depicts swimmer plot graphs for two groups: (A) 38 patients who showed positive T790M results from repeated tissue rebiopsies performed two or more times after disease progression, and (B) 56 patients who showed T790M positivity from repeated liquid rebiopsies. In this graph, the discontinuation point of first or second-generation TKI treatment indicated the time of disease progression. As demonstrated, even if T790M was initially negative after disease progression following the use of 1st line EGFR TKI, repeating the test at a different time point thereafter reveals the transition of T790M status from negative to positive over several months to years.

### 3.6 Response to 3^rd^ generation EGFR TKI between single rebiopsy and repeated rebiopsies

Among patients with confirmed T790M positivity, we analyzed 344 patients who used 3^rd^ generation TKI, comparing the response rates between those who underwent a single rebiopsy and repeated rebiopsies (**Supplementary Figure S3**). The Kaplan-Meier analysis demonstrates that patients with single rebiopsy exhibit a significantly longer median PFS of 14.3 months (95% CI, 11.9-17.0), compared to those undergoing repeated rebiopsies, who have a median PFS of 9.9 months (95% CI, 8.2-14.6) (log-rank p = 0.014). However, patients who underwent a single rebiopsy had significantly fewer extra-thoracic and brain metastases at the time of rebiopsy compared to those who had repeated rebiopsies. Additionally, there was no significant difference in ORR (49.6 vs. 43.3, *p*=0.402) and DCR (90.3 vs. 85.5, *p*=0.331) between the two groups (**Supplementary Table S4**).

## 4 Discussion

In this study, initial rebiopsy revealed a T790M positivity rate of approximately 40.8%, with subsequent repeated rebiopsies elevating this figure to 52.9%, demonstrating an overall increase of about 12.1%. Notably, among patients who initially tested negative for T790M, repeated rebiopsies uncovered an additional 23.5% T790M positivity. While the initial biopsy is critical for diagnosis, it does not guarantee complete detection of the T790M mutation. The increased detection rate through additional biopsies underscores the insufficiency of a single biopsy to fully determine a tumor’s mutational landscape (11–13). This study illustrates how the mutation profile of tumor varies over time and between different sites, indicating the need for repeated biopsies to accurately assess the presence of the T790M mutation (10).

Previous studies have shown that common EGFR mutations and longer duration of prior EGFR TKI treatment have an impact on T790M positivity (13–15). In addition, other studies have revealed that a larger tumor size and higher extra-thoracic disease burden are associated with a higher likelihood of positive plasma T790M results (16–18). Consistent with previous studies, this study also found that the group with a longer duration of previous EGFR TKI treatment and a better response to treatment significantly exhibited a higher positivity for the T790M mutation (**Table 2**; **Figure 2**). Additionally, while there was no difference between the groups in terms of the presence of extrathoracic metastasis (**Table 2**), it was observed that the rate of bone metastasis, which progresses slower and has a better prognosis compared to liver and CNS metastases (19–21), was significantly higher in the T790M positive group (**Table 2**; **Figure 2**). In repeated rebiopsy subgroup analysis, repeated tissue rebiopsy indicated that patients with longer durations of prior TKI treatment and better responses were more likely to show T790M positivity (**Supplementary Table S2**; **Supplementary Figure S1**). In contrast, liquid rebiopsies revealed a significantly higher likelihood of positivity in cases with bone metastasis (**Supplementary Table S3**; **Supplementary Figure S2**), thus demonstrating distinct characteristics between patients undergoing repeated tissue rebiopsies and those with repeated liquid rebiopsies. The higher rate of T790M positivity in plasma for patients with bone metastasis can be explained by several hypotheses. One such hypothesis is that bone metastases are often highly vascularized, which may lead to an increased shedding of tumor DNA into the bloodstream (22). Additionally, the microenvironment of bone metastases, characterized by active bone remodeling and increased vascular permeability, supports higher levels of ctDNA release. This process makes it more likely to detect mutations such as T790M in patients with bone metastases compared to those with metastases in other organs like the liver or brain (23).

The temporal heterogeneity of T790M status, as demonstrated by the transition from negative to positive in repeated rebiopsies was illustrated in **Figure 3**. It showed that the T790M status can change over time ranging from a few months to several years after the discontinuation of prior TKI treatment and disease progression. Previous studies also have reported that changes in EGFR mutations can occur over different time courses depending on a patient’s disease course and tumor burden (24–27). The change from a negative to a positive T790M result over time could be attributed to several factors. First, tumor evolution during the interval between tests may result in the emergence of the T790M mutation, leading to an initially true negative result that later becomes positive. Additionally, there is a possibility that an increase in tumor burden could result in an initially false negative result, as the mutation might not be detectable at lower tumor loads but becomes detectable as the tumor grows and the mutation-bearing cells become more prevalent (24, 28). This demonstrates the dynamic nature of the T790M mutation in response to treatment and the progression timeline, highlights the need for a flexible and adaptive approach to biopsy scheduling, tailored to the patient’s disease course and treatment response. Additionally, determining whether to perform tissue or liquid rebiopsies and selecting suitable patient groups for each method necessitates a strategic approach (29).

This study has several limitations. Being retrospective, it is challenging to discern clear criteria for the decision to perform repeated rebiopsies across different physicians. Additionally, it is difficult to ascertain detailed information on the timeline-specific mutation changes according to subsequent chemotherapy or immunotherapy. However, despite these limitations, this study analyzed a relatively large cohort of patients across multiple institutions, thereby provided insights on the expected patient demographics that could benefit from repeated rebiopsies in the real-world clinical setting.

In conclusion, this study demonstrated that among particular patient populations, the frequency of T790M positivity can be increased by conducting multiple rebiopsies. Notably, prolonged use of prior EGFR TKI, better responses to treatment, bone metastasis were closely associated with an increased likelihood of detecting T790M mutation. Therefore, in a clinical setting, for those patients exhibiting such characteristics, pursuing further repeated rebiopsies via tissue or liquid samples across varied sites and intervals can be considered, even when initial T790M testing results are negative.

## Data Availability

The original contributions presented in the study are included in the article/**Supplementary Material**. Further inquiries can be directed to the corresponding author.
