# Supplementary material for: Clinical utility of repeated rebiopsy for EGFR T790M mutation detection in non-small cell lung cancer
Source: Front Oncol. 2024 Aug 26;14:1452947. doi: 10.3389/fonc.2024.1452947 (PMC11381297; doi:10.3389/fonc.2024.1452947)
Supplement: Supplementary file 1 [file DataSheet1.docx]

**Supplementary Figure S1.** Forest plot for EGFR T790M positivity in patients with repeated tissue rebiopsies.


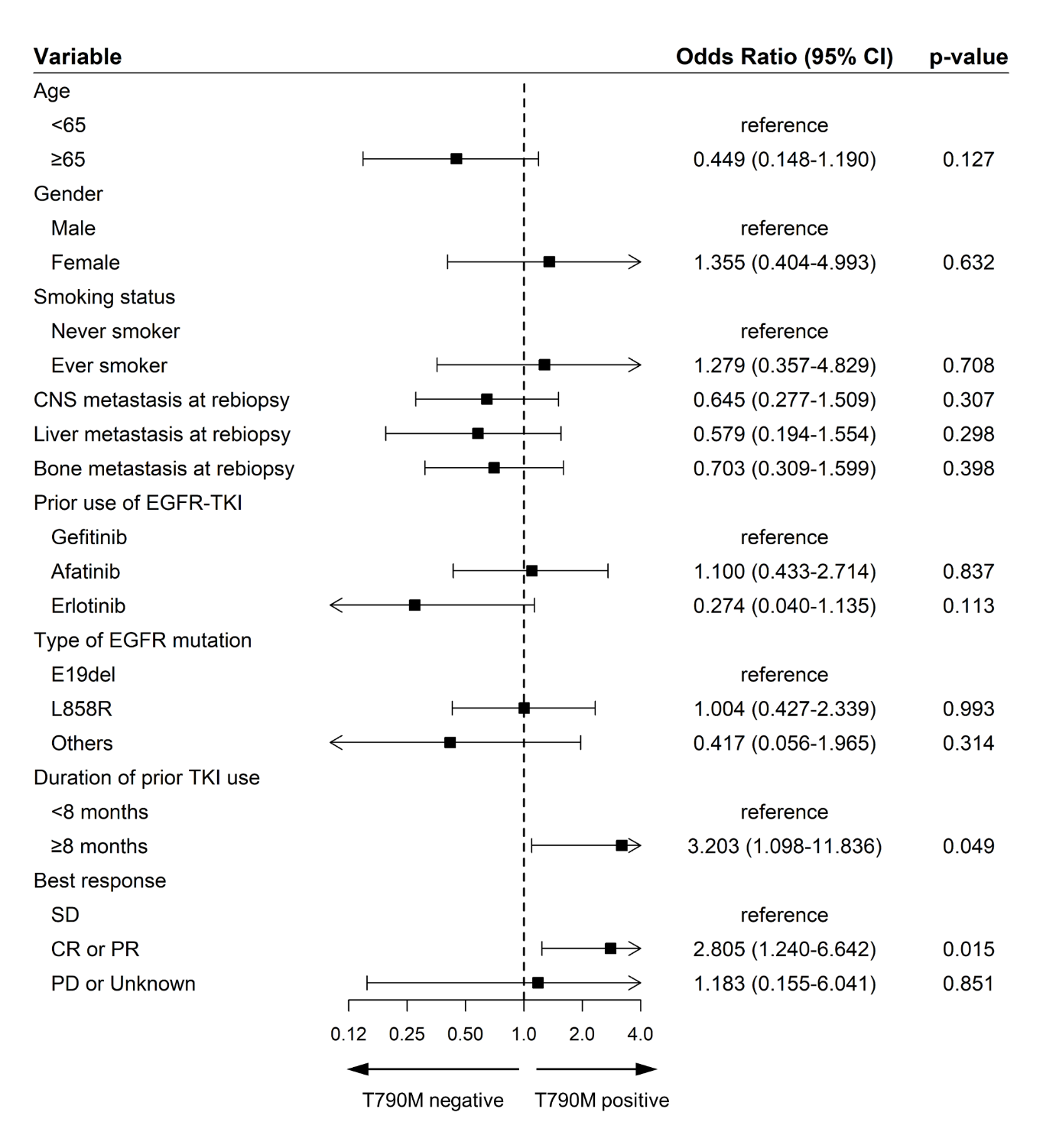


For patients who underwent repeated rebiopsy more than twice, the metastasis site immediately before the final biopsy was reflected. Squares represent odds ratios and bars 95% confidence intervals for the odds ratios. Features positively associated with EGFR T790M mutation show an odds ratio >1, whereas features negatively associated an odds ratio <1. OR, odds ratio; 95% CI, 95% confidence interval; EGFR, epidermal growth factor receptor; SD, stable disease; CR, complete response; PR, partial response; PD, progressive disease.

**Supplementary Figure S2.** Forest plot for EGFR T790M positivity in patients with repeated liquid rebiopsies.

**
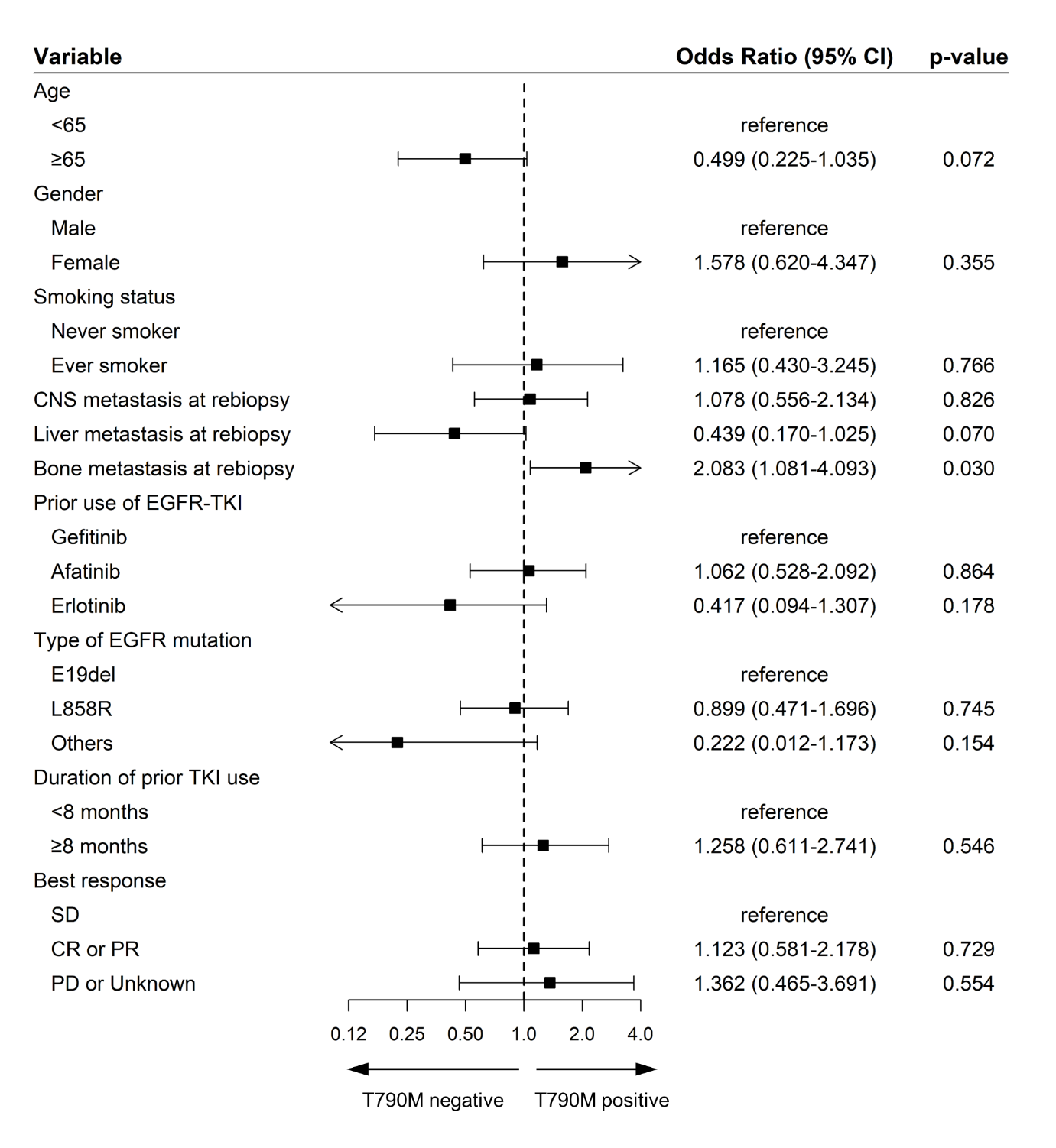
**

For patients who underwent repeated rebiopsy more than twice, the metastasis site immediately before the final biopsy was reflected. Squares represent odds ratios and bars 95% confidence intervals for the odds ratios. Features positively associated with EGFR T790M mutation show an odds ratio >1, whereas features negatively associated an odds ratio <1. OR, odds ratio; 95% CI, 95% confidence interval; EGFR, epidermal growth factor receptor; SD, stable disease; CR, complete response; PR, partial response; PD, progressive disease.

**Supplementary Figure S3.** Progression-free survival in patients treated with 3^rd^ generation EGFR TKI.


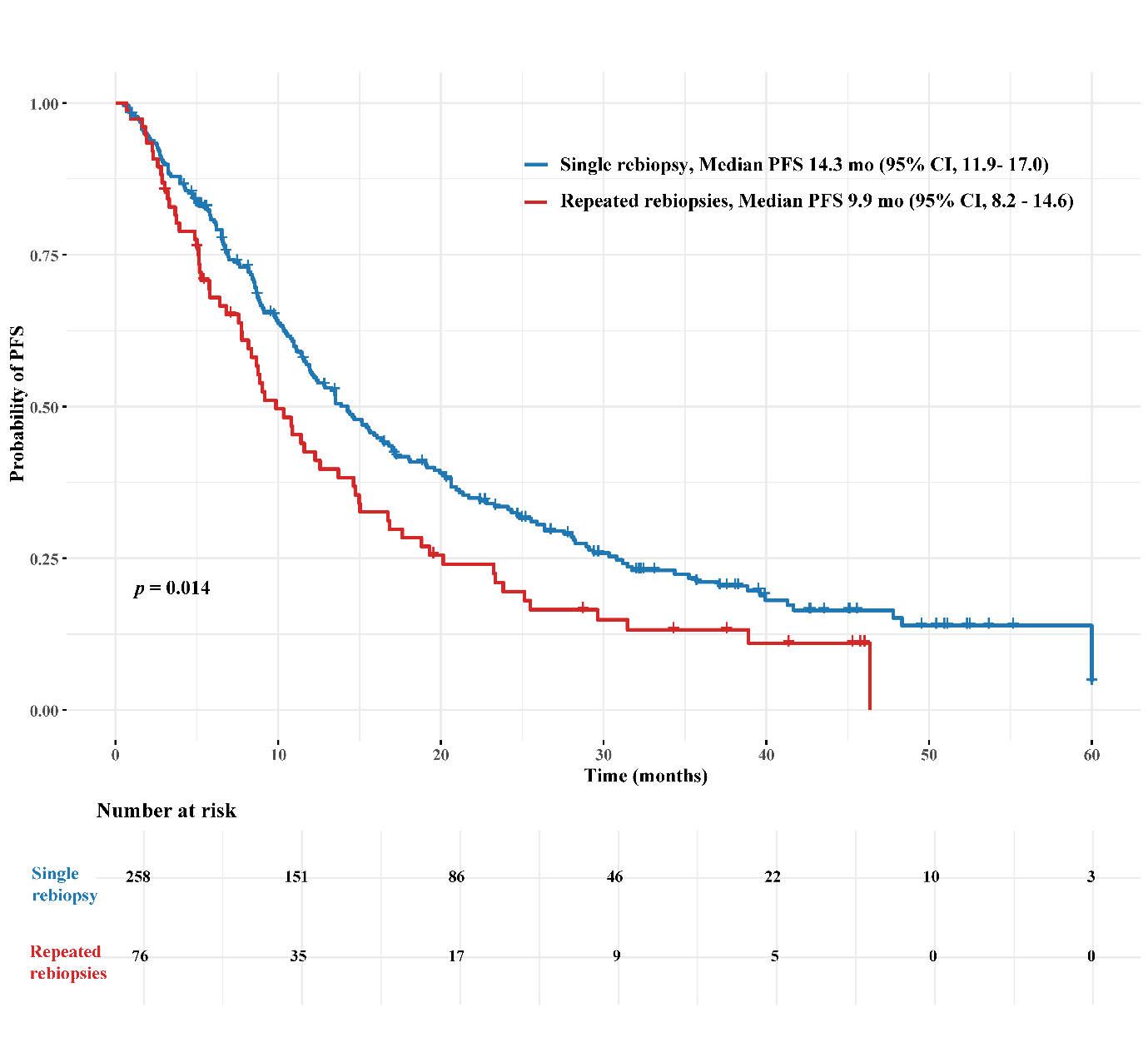


PFS, progression free survival; 95% CI, 95% confidence interval; EGFR, epidermal growth factor receptor; TKI, tyrosine kinase inhibitor.
