# Supplementary material for: Clinical utility of repeated rebiopsy for EGFR T790M mutation detection in non-small cell lung cancer
Source: Front Oncol. 2024 Aug 26;14:1452947. doi: 10.3389/fonc.2024.1452947 (PMC11381297; doi:10.3389/fonc.2024.1452947)
Supplement: Supplementary file 2 [file Table1.docx]

**Supplementary Table S1.** Tissue rebiopsy site and T790M positive rate.

| Rebiopsy Site | Total number | T790M positive number | T790M positive rate (%) |
| --- | --- | --- | --- |
| Lung | 342 | 138 | 40.4 |
| Metastatic LN | 163 | 61 | 37.4 |
| Pleural fluid | 19 | 9 | 47.4 |
| Liver | 29 | 9 | 31.0 |
| Brain | 10 | 2 | 20.0 |
| Bone | 9 | 3 | 33.3 |
| Others* | 16 | 7 | 43.8 |
| Overall | 588 | 229 | 38.9 |

*Other sites include omentum, skin, esophagus, chest wall, thyroid, pancreas, adrenal, kidney, glenoid mass etc.

**Supplementary Table S2.** Comparing patient characteristics based on EGFR T790M mutation status in repeated tissue rebiopsies.

| Characteristic | Total (N=165) | T790M-negative (N=127) | T790M-positive (N=38) | *P* value |
| --- | --- | --- | --- | --- |
| Age, median (IQR) | 59 (52.3-64) | 59 (53-65) | 56 (52-63) | 0.619 |
| ≥65 years, n (%) | 40 (24.2) | 34 (26.8) | 6 (15.8) | 0.166 |
| <65 years, n (%) | 125 (75.8) | 93 (73.2) | 32 (84.2) |  |
| Sex, n (%) |  |  |  |  |
| Male | 61 (37.0) | 49 (38.6) | 12 (31.6) | 0.433 |
| Female | 104 (63.0) | 78 (61.4) | 26 (68.4) |  |
| Smoking history, n (%) |  |  |  |  |
| Never smoker | 119 (72.1) | 91 (71.7) | 28 (73.7) | 0.633 |
| Ever smoker | 46 (27.9) | 36 (28.3) | 10 (26.3) |  |
| ECOG PS, n (%) |  |  |  |  |
| 0 | 69 (41.8) | 51 (40.2) | 18 (47.4) | 0.334 |
| 1 | 90 (54.5) | 70 (55.1) | 20 (52.6) |  |
| ≥2 | 6 (3.6) | 6 (4.7) | 0 (0.0) |  |
| Extra-thoracic metastasis at rebiopsy^a^, n (%) | 140 (84.8) | 108 (85.0) | 32 (84.2) | 0.99 |
| CNS metastasis at rebiopsy^a^, n (%) | 109 (66.1) | 86 (67.7) | 23 (60.5) | 0.531 |
| Liver metastasis at rebiopsy^a^, n (%) | 22 (13.3) | 16 (12.6) | 6 (15.8) | 0.814 |
| Bone metastasis at rebiopsy^a^, n (%) | 100 (60.6) | 79 (62.2) | 21 (55.3) | 0.563 |
| Prior use of EGFR-TKI, n (%) |  |  |  |  |
| Gefitinib | 99 (60.0) | 74 (58.3) | 25 (65.8) | 0.331 |
| Afatinib | 46 (27.9) | 35 (27.6) | 11 (28.9) |  |
| Erlotinib | 20 (12.1) | 18 (14.2) | 2 (5.3) |  |
| Type of EGFR mutation, n (%) | |  |  |  |
| E19del | 85 (51.8) | 64 (50.8) | 21 (55.3) | 0.693 |
| L858R | 65 (39.6) | 50 (39.7) | 15 (39.5) |  |
| Others^b^ | 14 (8.5) | 12 (9.5) | 2 (5.3) |  |
| Median duration of prior TKI use, month (IQR) | 13.0 (8.0-20.0) | 12.0 (7.0-21.0) | 14.4 (11.0-19.0) | 0.03 |
| ≥8 months, n (%) | 126 (76.4) | 92 (72.4) | 34 (89.5) | 0.03 |
| Best Response |  |  |  |  |
| CR | 1 (0.6) | 1 (0.8) | 0 (0.0) | 0.168 |
| PR | 76 (46.1) | 52 (40.9) | 24 (63.2) |  |
| SD | 77 (46.7) | 65 (51.2) | 12 (31.6) |  |
| PD or Unknown | 11 (6.7) | 9 (7.1) | 2 (5.3) |  |

^a^For patients who underwent repeated rebiopsy more than twice, the metastasis site immediately before the final biopsy was reflected. ^b^L861Q, S768I, G719X, Ex20Ins

**Supplementary Table S3.** Comparing patient characteristics based on EGFR T790M mutation status in repeated liquid rebiopsies.

| Characteristic | Total (N=291) | T790M-negative (N=235) | T790M-positive (N=56) | *P* value |
| --- | --- | --- | --- | --- |
| Age, median (IQR) | 60 (53-66) | 60 (54-68) | 57.5 (50-63) | 0.227 |
| ≥65 years, n (%) | 91 (31.3) | 80 (34.0) | 11 (19.6) | 0.037 |
| <65 years, n (%) | 200 (68.7) | 155 (66.0) | 45 (80.4) |  |
| Sex, n (%) |  |  |  |  |
| Male | 109 (37.5) | 92 (39.1) | 17 (30.4) | 0.222 |
| Female | 182 (62.5) | 143 (60.9) | 39 (69.6) |  |
| Smoking history, n (%) |  |  |  |  |
| Never smoked | 208 (71.5) | 168 (71.5) | 40 (71.4) | 0.058 |
| Ever smoker | 83 (28.5) | 67 (28.5) | 16 (28.6) |  |
| ECOG PS, n (%) |  |  |  |  |
| 0 | 136 (46.7) | 111 (47.2) | 25 (44.6) |  |
| 1 | 150 (51.5) | 119 (50.6) | 31 (55.4) | 0.484 |
| ≥2 | 5 (1.7) | 5 (2.1) | 0 (0.0) |  |
| Extra-thoracic metastasis at rebiopsy^a^, n (%) | 224 (76.9) | 176 (74.9) | 48 (85.7) | 0.121 |
| CNS metastasis at rebiopsy^a^, n (%) | 182 (62.5) | 144 (61.3) | 38 (67.9) | 0.447 |
| Liver metastasis at rebiopsy^a^, n (%) | 37 (12.7) | 30 (12.8) | 7 (12.5) | 0.957 |
| Bone metastasis at rebiopsy^a^, n (%) | 138 (47.4) | 104 (44.3) | 34 (60.7) | 0.038 |
| Prior use of EGFR-TKI, n (%) |  |  |  |  |
| Gefitinib | 170 (58.4) | 135 (57.4) | 35 (62.5) |  |
| Afatinib | 84 (28.9) | 66 (28.1) | 18 (32.1) | 0.182 |
| Erlotinib | 37 (12.7) | 34 (14.5) | 3 (5.4) |  |
| Type of EGFR mutation, n (%) | |  |  |  |
| E19del | 154 (52.9) | 121 (51.5) | 33 (58.9) |  |
| L858R | 116 (39.9) | 94 (40.0) | 22 (39.3) | 0.191 |
| Others^a^ | 21 (7.2) | 20 (8.5) | 1 (1.8) |  |
| Median duration of prior TKI use, month (IQR) | 13.0 (7.5-24.1) | 13.0 (7.0-24.6) | 13.5 (8.2-23.0) | 0.767 |
| ≥8 months, n (%) | 219 (75.3) | 175 (74.5) | 44 (78.6) | 0.523 |
| Best Response |  |  |  |  |
| CR | 4 (1.4) | 3 (1.3) | 1 (1.8) | 0.674 |
| PR | 124 (42.6) | 99 (42.1) | 25 (44.6) |  |
| SD | 136 (46.7) | 113 (48.1) | 23 (41.1) |  |
| PD or Unknown | 27 (9.4) | 20 (8.6) | 7 (12.5) |  |

^a^For patients who underwent repeated rebiopsy more than twice, the metastasis site immediately before the final biopsy was reflected. ^b^L861Q, S768I, G719X, Ex20Ins

**Supplementary Table S4.** Comparison between single rebiopsy and repeated rebiopsies patients among T790M positive cases

|  | Single rebiopsy (n=258) | Repeated rebiopsies (n=76) | p-value |
| --- | --- | --- | --- |
| Age, median (IQR) | 64 (55 - 71) | 57.5 (51-63) | <0.001 |
| Sex, male, n (%) | 98 (37.9) | 23 (30.3) | 0.273 |
| Prior use of EGFR-TKI, n (%) |  |  |  |
| Gefitinib | 157 (60.8) | 50 (65.8) | 0.035 |
| Afatinib | 58 (22.5) | 22 (28.9) |  |
| Erlotinib | 43 (16.7) | 4 (5.3) |  |
| Type of EGFR mutation, n (%) |  |  |  |
| E19del | 164 (63.6) | 42 (55.3) | 0.22 |
| L858R | 86 (33.3) | 33 (43.4) |  |
| others | 8 (3.1) | 1 (1.3) |  |
| Median duration of prior 1^st^ or 2^nd^ generation TKI use, month (IQR) | 15 (9-27) | 14 (9.2-21) | 0.311 |
| Extra-thoracic metastasis at rebiopsy^a^, n (%) | 166 (64.3) | 64 (84.2) | 0.002 |
| CNS metastasis at rebiopsy^a^, n (%) | 99 (38.4) | 49 (64.5) | <0.001 |
| Liver metastasis at rebiopsy^a^, n (%) | 29 (11.2) | 10 (13.2) | 0.799 |
| Bone metastasis at rebiopsy^a^, n (%) | 136 (52.7) | 45 (59.2) | 0.385 |
| 3^rd^ generation TKI |  |  |  |
| Osimertinib | 214 (82.9) | 63 (82.9) | 0.988 |
| Lazertinib | 40 (15.5) | 12 (15.8) |  |
| Both | 4 (1.6) | 1 (1.3) |  |
| Best response to 3^rd^ generation EGFR TKI |  |  |  |
| Partial response | 128 (49.6) | 33 (43.4) | 0.121 |
| Stable disease | 105 (40.7) | 32 (42.1) |  |
| Progressive disease | 6 (2.3) | 6 (7.9) |  |
| Unknown | 19 (7.4) | 5 (6.6) |  |
| ORR of 3^rd^ generation EGFR TKI | 128 (49.6) | 33 (43.3) | 0.402 |
| DCR of 3^rd^ generation EGFR TKI | 233 (90.3) | 65 (85.5) | 0.331 |

*Among the 361 T790M positive patients, 27 were unable to use 3^rd^ generation TKI or were lost to follow-up, resulting in a total of 334 patients analyzed in this table.

^a^For patients who underwent repeated rebiopsies more than twice, the metastasis site immediately before the final biopsy was reflected. ORR, objectiver response rate; DCR, disease control rate.
